# Harvard Odontological Society

**Published:** 1894-08

**Authors:** 

**Affiliations:** Harvard Odontological Society


### HARVARD ODONTOLOGICAL SOCIETY.

At a regular meeting of the Harvard Odontological Society, held March 29, 1894, at Young's Hotel, Boston, President Eddy in the chair, a paper was read on "Some Observations on the Extraction of Teeth to prevent Decay," by Dr. Eugene H. Smith.

(For Dr. Smith's paper, see page 481.)

### DISCUSSION.

*Dr. Werner.*—Mr. President, I know of no operation where it is more necessary to have clear perception and good judgment than in that which Dr. Smith has just described. Generally speaking, when there is a question of extraction the operator should possess clear judgment, because he is burning his bridges. In all the cases that the essayist has shown, it is evident that he has exhibited this clear conception. It certainly lessens decay, and it seems in almost every case to have improved the condition and position of the third molar, and that is very important. Many of our patients have an idea that the third molar is a very poor tooth, but we are all familiar with that error, and in observing cases where the first molar has been extracted, one can readily see what a useful position the third molar occupies. The time of extraction also calls for good judgment. The only thing the models to my mind show, where the least adverse criticism can be applied, is in the considerable space left between the occluding surfaces of the teeth. Extraction, of course, destroys the continuity of grinding surface; but that, in a healthy mouth, is of much less consequence than extensive decay. However, in these models I do not know that I have anything to say against it, as they show that it was undoubtedly the wise thing to do. It has certainly helped to lessen the tendency towards decay, and also towards irregularity, and it has improved the condition and position of the third molar. Old Dr. Tucker is often spoken of as having said that it is better to have eight teeth in the mouth in good condition, than to have sixteen in poor condition. That is only expressing in strong terms the desirability of extraction in some cases.

*Dr. Cooke.*—Mr. President, I remember hearing Dr. Davis, of New Bedford, several years ago, take the ground that the extraction of the twelfth-year molars as soon as they came through prevented the decay between the teeth, and also gave the wisdom-tooth a chance to develop so that it would be of service. I would like to ask Dr. Smith what he thinks would be the result in a case where we are able to preserve the sixth-year molar and keep it in good condition,—whether he thinks it would be good practice to take out the four twelfth-year molars for the purpose of preventing this decay between the bicuspids and the sixth-year molars and the crowding forward of the incisors?

*Dr. Smith.*—My observation is, Mr. President, that at that time, when the sixth-year molars are well in place and established,—that is, when they have been there for six or seven years and are firmly embedded in the jaw,—that I question whether you could obtain any great movement posteriorly of those teeth. The tendency to move is anteriorly. We often see that later in life, when a child, who in the teens presented a perfect æsthetic line, will show a few years later the lower central incisors and lower lateral incisors overlapping. Of course, this is due in a measure to the erupting of the lower wisdom-teeth, and yet in many instances where the twelfth-year molar or the wisdom-tooth is extracted you still have that overlapping. The case which you cite and the advice given by Dr. Davis may have been largely influenced by circumstances; but where the object sought is to prevent this lateral impingement, I

should prefer to take out the second bicuspid if the other teeth were good. We are forced sometimes, especially in infirmary work, to do things which may not be in accord with our own wishes.

*Dr. Cooke.*—Is the essayist of the opinion that the decay between the bicuspid and the molar is due simply to food which is lodged there, or is that decay increased by pressure,—by the force that is exerted during the process of mastication? Is that a condition which of itself will produce more decay?

*Dr. Smith.*—I think that it has an influence; how much I do not know. The main point is to have these teeth drop back as far as possible, so that we can have them self-cleansing, permitting the free working of the saliva and fluids about the teeth. With such a condition, of course, the secretions and fluids are not held there. That is the main object in extracting. How much this real power of pressure increases the progress of decay in places which are already favorable to its advancement I am not able to say, but the closer the teeth are together the more difficult it is to get anything which is lodged between the teeth removed, and, therefore, decay must increase in the same ratio.

*Dr. Briggs.*—I think that what the essayist has said is in accord with my opinion of the subject, and I can only endorse his remarks. I was first brought up in the school, or at least under a preceptor who was very skilful in the use of the forceps and took out teeth very liberally. Later on, I came under the influence of too much conservatism; everything was saved, and it took me some time to emancipate myself from that into what I think now is a happy medium, where I do take out teeth for the sake of the other teeth in the mouth. I am sometimes confronted with a greater problem, perhaps, than any which has been presented here, in that the question comes of taking out better teeth than the essayist has shown. In some of the cases that he has shown the teeth have been so bad that it was not a difficult task to settle the question, but there are times when one takes out very good teeth in order to bring the whole mouth into a better hygienic condition. I think that there is sometimes a necessity for a word of caution and advice to practitioners who have started out with the idea of saving all the teeth, to prevent them from going too far in that direction. Only last week I had an example of this in a case that was sent to me from another practitioner, where the upper wisdom-teeth were in very bad condition. They had no opponents, no usefulness in the mouth, and yet they had been kept along and treated with soft-fillings. They were out of the reach of the brush and were not cleanly, and as a consequence the whole mouth was affected by them; and it seems to me in such cases very foolish to insist on trying to keep such teeth in the mouth. The last case of this nature that I had was very interesting, in that the patient had some trouble from facial paralysis,—evidently some irritation which may have passed away and left the parts benumbed; or the teeth may have been blameless, but I found the chamber in the third molar on that side after extraction entirely filled with pulp-stone, which may have been the cause of the irritation. That tooth had been kept for years, keeping the whole mouth in an unkempt condition and serving no usefulness whatever.

As I say, however, when you have a case from childhood and have watched the progress of the permanent teeth, the sixth-year molars do not get in a very bad condition, and I find it more expedient in such cases, and it is my custom, to take out the bicuspids. My own experience and clinical observations have led me to feel that, other things being equal, the sixth-year molar is the better tooth to keep, because if you carry a sixth-year molar up to the age of twenty, you can generally carry it on, under favorable conditions, during life; in other words, it is apt to improve. Bicuspids, however, seem to begin at about twenty to deteriorate rapidly, and you are apt to get rapid decay or splitting; so I try as far as possible to make it appear that it is a bicuspid which should come out instead of a molar.

HENRY L. UPHAM, D.M.D.,
*Editor Harvard Odontological Society.*

